# Recent advances on CAR-T signaling pave the way for prolonged persistence and new modalities in clinic

**DOI:** 10.3389/fimmu.2024.1335424

**Published:** 2024-02-22

**Authors:** Sergei Smirnov, Polina Mateikovich, Konstantin Samochernykh, Evgeny Shlyakhto

**Affiliations:** Almazov National Medical Research Centre, Personalized Medicine Centre, Saint Petersburg, Russia

**Keywords:** CAR (chimeric antigen receptor), persistence, activation, costimulatory, signaling, T cell, immunotherapy

## Abstract

Chimeric antigen receptor T-cell (CAR-T) therapy has revolutionized the treatment of hematological malignancies. The importance of the receptor costimulatory domain for long-term CAR-T cell engraftment and therapeutic efficacy was demonstrated with second-generation CAR-T cells. Fifth generation CAR-T cells are currently in preclinical trials. At the same time, the processes that orchestrate the activation and differentiation of CAR-T cells into a specific phenotype that predisposes them to long-term persistence are not fully understood. This review highlights ongoing research aimed at elucidating the role of CAR domains and T-cell signaling molecules involved in these processes.

## 1 Introduction

CARs provide the T-cell with a targeting system that enables it to recognize antigens on the surface of a tumor cell in an HLA-unrestricted manner and to orchestrate its cytotoxic functions. The functional properties of CAR-T cells are determined by the structure of the chimeric antigen receptor, the components of which are not only responsible for cytotoxicity against target cells, but also influence phenotype and persistence (1, 2).

**Figure 1:**
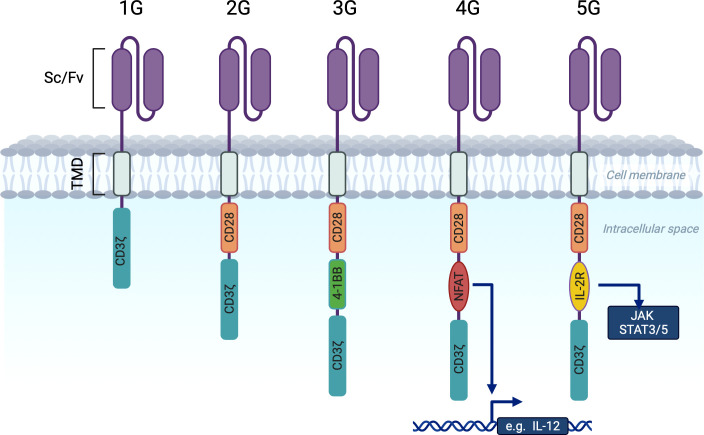
Chimeric antigen receptor generations. scFv (single chain variable immunoglobulin fragment). TMD (Transmembrane domain (controls membrane integration and expression level. Most often derived from CD8α or CD28). 1G CARs contain only the CD3ζ activating molecule in the intracellular domain. 2G and 3G CARs contain one or two costimulatory domains (CD28 and 4-1BB) in addition to CD3ζ. 4G CARs have a costimulatory domain paid with an NFAT responsive cassette that activates chemokine expression upon CAR activation (e.g. IL12 or IL18). 5G CARs are based on 2G with the addition of a truncated intracellular interleukin 2B chain receptor providing a STAT3 binding motif and a STAT5 binding motif (not shown).

The first generation (1G) of chimeric antigen receptors (CARs) consisted of an extracellular antigen recognition domain (single-chain variable immunoglobulin fragment) linked to an intracellular activation domain (CD3ζ) by a transmembrane domain (**Figure 1**) (3, 4). However, first generation CARs had limited persistence (5).

The incorporation of a costimulatory domain into the CAR construct paved the way for second-generation (2G) dual-signaling CARs (6). These have improved persistence and anti-tumor functions *in vivo*. The most widely used costimulatory domains in 2G CAR-T cells are CD28 (6–8) and 4-1BB (9, 10). We will refer to CD19-targeting 2G CAR-T cells with these domains as 19-28ζ and 19-BBζ.

More recently, it has been recognized that these 19-28ζ and 19-BBζ differ in many aspects (discussed below) and researchers have concluded that it is reasonable to combine both domains in one CAR (11), that had led to the emerge of the third generation of CAR-T (3G). Besides the above, 3G CAR-T cells used other costimulatory domains (12, 13).

Fourth generation CAR-T cells are based on 2G CAR-T cells with the addition of an NFAT (nuclear factor for activated T cells) responsive cassette. This cassette regulates the expression of transgenic proteins (IL-7, IL-12, IFN-γ) that are inducible upon CAR-T cell activation (14, 15). These 4G CAR-T cells are also known as T-cells redirected for universal cytokine-mediated killing (TRUCK). It is known that T cells need to receive three signals to be fully activated: an activation signal via the CD3ζ molecule, a costimulatory signal (from the costimulatory domain) and a third signal of cytokine engagement. The expression of cytokines promotes the activation of innate immunity (14) and may contribute to the modulation of the tumor microenvironment (16).

Fifth generation (5G) CAR-T cells, also known as the next generation, were generated by adding a truncated cytoplasmic domain from the interleukin IL-2 receptor B chain IL-2RB (a costimulatory domain associated with activation of the transcription factor STAT5) between the cytoplasmic domains of CD28 and CD3ζ, and a STAT-3 binding motif (YXXQ) at the C-terminus of CD3ζ (17). Upon contact with the antigen, a cell with such a receptor receives three activation signals simultaneously from the CD3ζ domain, the costimulatory domain (CD28) and the JAK-STAT 3/5 pathway, which promotes CAR-T cell proliferation and persistence.

While the 1G CARs have been generated empirically (18) and the 2G has been successfully used in the clinic (19), intracellular signaling in CAR-T cells is still not fully understood. On the one hand, the degree of activation of CAR-T cells is crucial for the successful eradication of malignant cells. On the other hand, excessive activation of CAR-T cells can lead to uncontrolled systemic inflammation, cytokine release syndrome (20) and the rapid onset of the exhaustion phenotype of CAR-T cells (21). Therefore, an important step for successful therapy is to comprehensively control CAR activation and costimulation.

The plethora of clinical trials has shown that the durability of clinical remissions in patients with chronic and acute lymphocytic leukemia is strongly correlated with the persistence of CAR-T cells (22, 23). The result of CAR-T therapy dramatically depends on the quality of initial aphaeresis products. According to the results of at least 100 CLL (chronic lymphocytic leukemia) and ALL (acute lymphocytic leukemia) patients treated with CAR-T frequency of naїve-like T-cells within the starting aphaeresis is the major factor influencing outcome of the therapy (24). Notwithstanding this limitation, there are several strategies to prolong the *in vivo* persistence and efficacy of CAR-T cells. In particular, modulation of the culture media or simply limiting the culture time has been reported to improve engraftment and effector function by maintaining higher levels of memory CAR-T cells (24). What’s more, it is becoming increasingly apparent that modulation of CAR signaling, together with other strategies to prolong the *in vivo* persistence, has the potential to broaden the scope of immunotherapy and pave the way for its application beyond hematological malignancies.

Herein, we review recent advances in the research of CAR-T cell singling that have the potential to overcome the limited persistence and excessive activation of CAR-T cells. Engineered cytokine singling in so-called ‘armored’ CARs (25) has been reviewed elsewhere (26) and is not the focus of this review.

## 2 Proximal and distal CAR-T cell signaling

**Figure 2:**
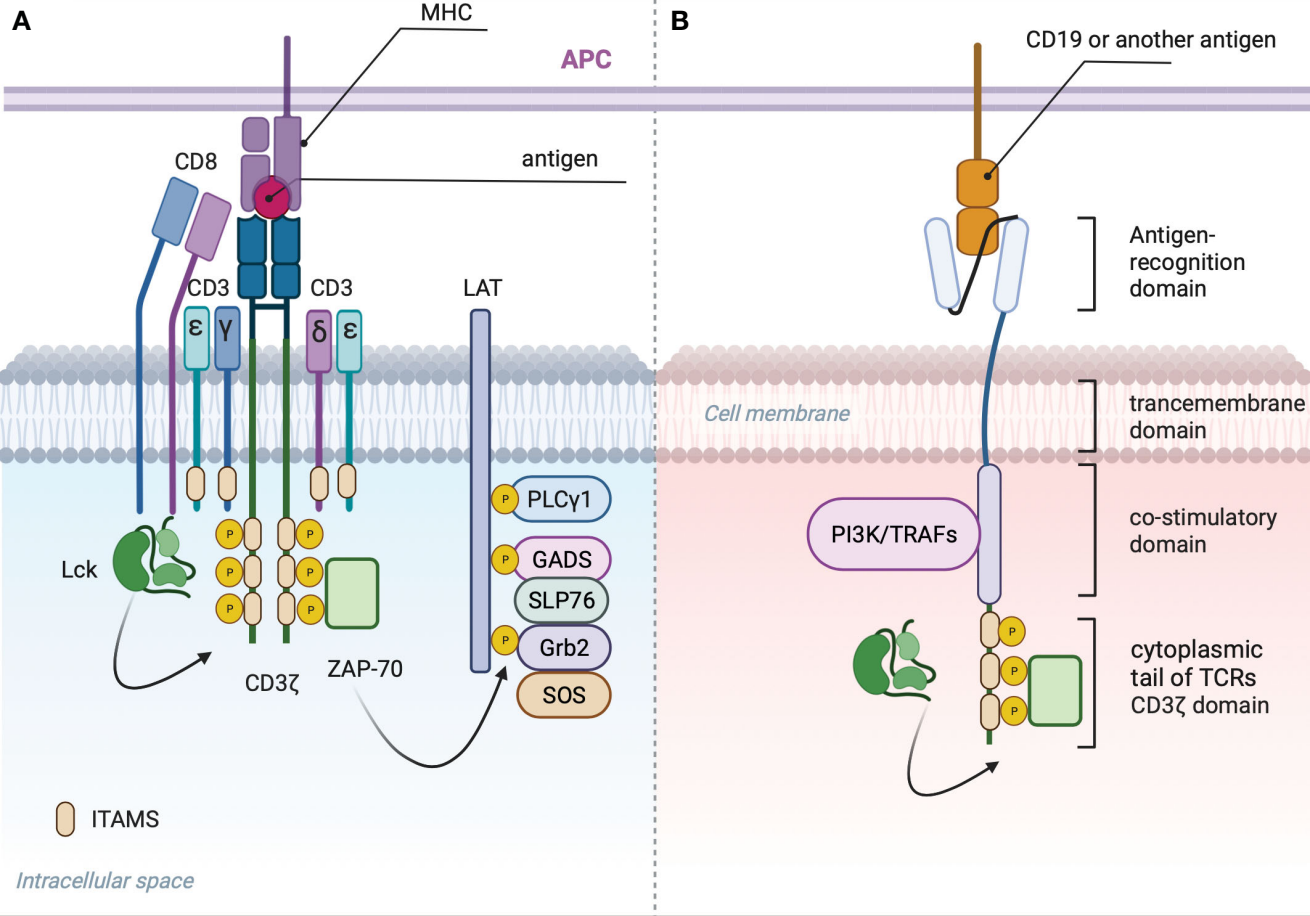
**(A)** TCR signalosome: Phosphorylation of two ITAMS within CD3ζ domain by LSK provides scaffold for ZAP-70 that in activated state dissociates and phosphorylates linker of activated T cells (LAT) (27). Phosphorylated LAT provides scaffold for growth-factor-receptor-bound protein (GRB2) that associates with son of seven less homologue (SOS) with subsequent activation of RAS-ERK (extracellular-signal-regulated kinase) signaling, GRB2-related adaptor protein (GADS) that binds with the adaptor protein SLP76, PLC-γ1 and other proteins (28). Phosphorylated SLP76 and PLC-γ1 via several intermediates induces an increase in the cytoplasmic calcium concentration (29) and activation of nuclear factor of activated T cells (NFAT). **(B)** General CAR signaling: Phosphorylated CD3ζ recruits Zap70 resulting in Ca influx and translocation of NFAT to the nucleus. CD28 costimulatory domain recruits PI3K, additional Lsk and GRB2, resulting in AKT, NF-kB, AP-1 and mTOR signaling activation. 4-1BB recruits TRAFs with subsequent activation of NF-κB AP-1 and ncNF-κB pathways.

T cell receptor is composed of an antigen recognition subunit - TCRαB (**Figure 2A**) and signaling subunits: CD3ζζ, CD3ϵδ and CD3ϵγ (30). The endogenous TCR complex mediates antigen-induced signaling through 10 immunoreceptor tyrosine-based activation motifs - ITAMS (31). Each CD3ϵδ and CD3ϵγ subunit have 2 ITAMS, whereas dimer CD3ζζ have 6 ITAMS (32, 33). CARs have only 3 ITAMS within CD3ζ domain or 6 if they form dimers (**Figure 2B**). Downstream consequences of antigen recognition by TCR or CAR is the phosphorylation of ITAMs within CD3ζ (34) domains by the lymphocyte-specific protein tyrosine kinase (LCK). Dual phosphorylation of ITAMS within CD3ζ enables interaction with SH2 tandem domains of Zeta-chain-associated protein kinase 70 (Zap70) with a consequent release of the last from autoinhibited conformation and activation of the downstream signaling cascade that predispose the effector functions of T-cells (35). Phosphorylation of only one ITAM within CD3ζ causes minimal binding with Zap70 (36).

Two the most frequently used costimulatory domains for CARs are CD28 and 4-1BB derive from CD28 family and the tumor necrosis factor receptor (TNFR) family. Particularly the first two FDA approved CAR-T Kymriah and Yescarta incorporates a 4-1BB and CD28-derived costimulatory domains respectively while sharing the same scFv that binds CD19.

Despite CD28 lacks ITAMs that disable its ability to recruit ZAP-70 (37), it could functionally mimics LAT by recruiting phosphatidylinositol 3-kinase (PI3K) (38) with subsequent AKT, canonical NF-kB and mTOR signaling activation. Furthermore, CD28 can recruits growth factor receptor-bound protein 2 (GRB2) (39) that in turn activates Ras and ERK (40). Transcriptome analysis showed up-regulation of PI3K/AKT and glycolysis pathways in CD33 CAR-T bearing CD28 compared to control T cells (41). The authors also pointed to an antigen-independent constitutive activation of PI3K that reduced the *in vivo* persistence of CAR T cells.

Downstream signaling molecules recruited by 4-1BB are tumor necrosis factor receptor-associated factors (TRAFs) that activate nuclear factor kappaB (NF-κB) (42). Gongo Li et al. demonstrated that enhanced persistence of CD19-targeted CAR-T cells with 4-1BB costimulatory domain is driven by NF-κB (43). In addition, 4-1BB domain have the capacity to activate non-canonical nuclear factor κB (ncNF-κB) pathway (44) that is known to promote T-cell survival in diverse context (45, 46), thereby enhancing the expansion and survival of CAR-T cells.

## 3 19-28ζ and 19-BBζ cells in preclinical studies

The kinetics of tumor elimination was studied in the NALM/6 ALL model (47) to compare the contribution of the CD28 and 4-1BB domains to therapeutic efficacy (11). CAR-T cells with CD28 costimulatory domain were able to achieve faster tumor eradication within the first 7 days when the dose was reduced to levels below the therapeutic dose (4×10^5^, 2×10^5^ and 1×10^5^ CAR-T cells). The number of 19-BBζ cells exceeded the number of 19-28ζ by day 14 after administration, indicating a higher proliferative capacity of cells with this domain, but comparable elimination of tumor mass was not achieved.

However, in a more recent study (21) in a xenograft mouse model of lymphoma, 19-BBζ showed to be more effective when administered at low dose (8 × 10^5^ cells). This disparity may be explained by differences in CAR structure except for costimulatory domain. Particularly in the work of A. Salter et al. a portion of CD28 was used as a hinge and transmembrane region (H/TM) in both constructs, which has been shown (48) to result in higher levels of CD19-specific CD3ζ phosphorylation and higher quantities of released IFN-γ, TNF-α and IL-2. In the context of increased amplitude of activation, 19-BBζ may have gained an advantage over 19-28ζ. On the contrary, H/TM from the CD8α gene used in the work of Z. Zhao et al. decreased the activation threshold (48) and created certain advantages for 19-28ζ.

Phosphoproteomics analysis of 19-28ζ and 19-BBζ CARs signaling showed that more intense 19-28ζ CAR-mediated signaling correlated with higher levels of PD-1 expression and the exhausted phenotype (21). The increased signaling was partially associated with constitutive LCK association with the CD28 domain. However, LCK was shown to be predominantly recruited to CAR by the CD-8 and CD-4 co-receptors rather than the CD28 domain of CAR (49). Stimulation via CD28/CD3 or 4-1BB/CD3 led to phosphorylation of almost identical proteins, but the main differences were in the dynamics of this phosphorylation. Thus, CD28/CD3 stimulation led to faster and more intense phosphorylation of proteins involved in the T-cell activation cascade and in the forming of effector phenotype. Differentially expressed gene analysis showed increased expression of genes associated with memory T-cell formation (KLF2 and IL7R) in 19-BBζ. Co-immunoprecipitation of CARs showed that there was a stronger interaction between the THEMIS (thymocyte expressed molecule involved in selection) phosphatase SHP1 complex and 19-BBζ, when compared to 19-28ζ. The knockdown of either THEMIS or SHP1 in 19-BBζ cells increased CD3ζ phosphorylation. Thereby the authors arrived into the conclusion that the THEMIS-SHP1 phosphatase complex attenuates CD3ζ phosphorylation in 19-BBζ.

More recently, 19-28ζ and 19-BBζ cells have been shown to differ in their antigen sensitivity (50). At a low dose (0.2 × 10^6^ cells), both types of CAR-T cells failed to prevent the growth of NALM6 acute lymphoblastic leukemia (ALL) tumor cells in an immunodeficient mouse. Moreover, at the time of relapse, 19-BBζ cells were markedly exhausted and 19-28ζ were undetectable. CD19 expression on the surface of NALM6 cells at the time of relapse was unchanged in samples treated with 19-28ζ, but decreased significantly (from 11,000 to 4,500 molecules) in samples treated with 19-BBζ. When target cells were co-cultured with T-cells or with T-cells expressing non-functional CAR, there was no reduction in CD19 expression. After co-culturing CAR-T cells with target cells expressing a CD19 molecule cross-linked to the fluorescent protein mCherry, both the fluorescent protein and the CD19 molecule were detected on the CAR-T cells. Thereby, the authors demonstrated that CD19 molecules are transferred from the target cell to the CAR-T cell by CAR-mediated trogocytosis. CAR-T cells expressing CD19 were susceptible to T-cell fratricidal killing and expressed markers of depletion (PD-1, LAG-3 and TIM-3). It is also worth noting that administration of a new dose of 19-BBζ cells to mice with recurrent tumor growth did not lead to tumor reduction, in contrast to repeated infusions of 19-28ζ cells. The latter showed that the number of CD19 molecules expressed on target cells fell below the threshold required to activate 19-BBζ. The strategy of targeting 2 antigens and combining of 28ζ and BBζ costimulatory domains showed the best results. In particular, treatment of mice with established NALM6_med_ and NALM6_low_ with a low dose (0.2 × 10^6^) of 19-28ζ and 22-BBζ combination prolonged response and survival in all mice.

Judith Feucht et al. sought to impede the phosphorylation of tyrosine residues within CD3ζ by generating single ITAM containing mutants (51). Ablation of N-terminal ITAMS in CARs with CD28 costimulatory domain (XX3) had led to diminished anti-tumor efficacy, whereas CAR-T cells with C-terminal mutated tyrosine residues (1XX) outperformed the CARs without mutation. These cells exhibited higher persistence and elevated levels of central memory T cells (CD62L+CD45RA−). Gene set enrichment analysis (GSEA) emphasized down-regulation of T cell activation and effector-related genes in 1XX CARs relative to CARs without mutations. Thereby, the authors showed that number and position of ITAMS plays a crucial role in CAR functions.

Apparently, mutations in the CD3ζ domain, that improve the therapeutic effect of 19-28ζ by reducing the amplitude of activation of such cells and subsequent exhaustion, would not play such a role in 19-BBζ. On the contrary, the sensitivity of such cells to antigen and their activation and proliferation in response to antigen stimulation would have been reduced by further dampening of 19-BBBζ activation. Recent studies in a mouse model showed that CAR-T cells with only one active membrane-proximal ITAM (1BBζ**) had reduced ability to inhibit CD19_low_ NALM6 cell growth (52). By adding an additional CD3ζ molecule (CD19-4-1BBζζ), the researchers sought to increase the intensity and amplitude of activation of 19-BBζ cells in response to lower antigen densities (52). The increased levels of phosphorylated CD3ζ and ERK molecules were indicative of an increased amplitude of activation; at the same time, the authors did not observe an increase in the expression of exhaustion markers (PD1, TIM-3 or LAG3) on these cells. In vitro, CD19-4-1BBζζ cells showed an ability to suppress the growth of tumor with a low CD19 density comparable to that of 19-28ζ and a significantly higher persistence.

Among other CD3 domains within TCR the CD3ϵ ITAM has the lowest affinity to Zap70 (53). This domain is mono-phosphorylated by LCK upon TCR stimulation and recruits inhibitory kinase Csk (54). In addition CD3ϵ utilizing the basic residue rich sequence (BRS) can recruit p85, the regulatory subunit of PI3 kinase (PI3K) that is an upstream regulator of AKT. It is worth mentioning that AKT serine/threonine kinases are crucial mediators of the proliferation and survival of T-cells (55, 56). In a recent publication, CD3ϵ domain was incorporated in the second generation CAR (57) N-terminal to CD28 costimulatory domain (54). In a xenograft mouse model of lymphoma, the resulting CD19.E28Z CAR-T cells, while producing significantly lower levels of cytokines (IL-2, IFN-γ, TNF-α), had an increased proliferative capacity and a greater ability to suppress tumor growth than cells with control CARs developed by James N. Kochenderfer in his well-known work (57). Overall the authors arrived at the conclusion that activation of TCR signaling by LCK and its attenuation by Csk is a self-restrained signaling machinery (54). In addition, the incorporation of CD3ϵ into CAR construct enhances CAR-T cells persistence via accelerating AKT signaling on one hand, and diminishing its proximal signaling cascade via recruitment of inhibitory kinase on the other hand.

Another study investigated the differential persistence of CAR-T cells (58). More specifically, the assessment of CD45RO and CCR7 surface markers expression revealed the enrichment of stem-like memory cell markers on 19-BBζ. In contrast, 19-28ζ showed a higher proportion of effector memory cell phenotype (CD45RO+CCR7- cells). The development and persistence of naive and memory T-cells rely on mitochondrial oxidation of fatty acids (59), whereas effector T cells switch to glycolysis (60). The authors speculated that signaling from the 4-1BB domain supports mitochondrial biogenesis. Further experiments showed increased levels of certain mitochondrial genes, confirming this hypothesis. These were mitochondrial transcription factor A (TFAM), mitochondrially encoded cytochrome c oxidase 1, nuclear respiratory factor 1 (NRF1) and GA-binding protein (NRF2).

## 4 19-28ζ and 19-BBζ cells in clinical studies

Despite differences in the activation of pathways, both costimulatory domains have shown comparable efficacy (61, 62) in achieving high rates of complete remission in patients with some forms of B-cell malignancies.

However, relapses and frequent serious side effects such as cytokine release syndrome have also been observed with both costimulatory domains (63, 64). It is difficult to assess the effect of these domains on therapeutic efficacy based on the results of numerous studies using one or the other domain, due to structural differences other than the costimulatory domains in the receptors used (hinge/transmembrane domain) and differences in the production process of CAR-T cells (63, 64).

Clinical trials of 19-BBζ in pediatric and young adults with relapsed/refractory B-cell acute lymphoblastic leukemia (B-ALL) revealed that durable remission is sustained when CAR-T cells persists at least for several months (65) and 4-1BB costimulation appeared to contribute to prolonged persistence (66).

The disparity between the therapeutic efficacy of 19-BBζ (Kymriah or CTL019) in patients with chronic lymphocytic leukemia (durable response 26%) and relapsed or refractory acute lymphoblastic leukemia (complete remission 90%) was highlighted by Joseph A. Fraietta et al. The authors elaborate on T-cell intrinsic mechanisms that predispose to durable anti-tumor effects (67). Long-term remission was associated with the enrichment of the CD27+CD45RO-CD8+ T cell memory phenotype among adoptively transferred CAR-T cells. Transcriptomic profiling of CAR-T cells from fully responding and non-responding patients revealed up-regulation of memory-related genes (IL-6/STAT3 signatures) in the former and enhanced expression of genes involved in effector differentiation, exhaustion and glycolysis in the latter.

While the achievement of complete responses and prolonged remissions with 19-BBζ was clearly associated with prolonged persistence (2-9 years) (65, 68), the use of 19-28ζ in the treatment of diffuse large B-cell lymphoma and follicular lymphoma showed efficacy with relatively low (compared to 19-BBζ) persistence (69). The highest number of CAR+ cells in the blood was detected on days 6-35 and decreased to 0-1 cells per 1μl by 3 months in all patients. At the same time, the authors noted the association of complete and partial responses to therapy with higher peak blood concentrations of CAR+ cells. In a more recent publication reflecting the long-term follow-up results of this clinical trial, it was noted that the achievement of a durable response to therapy (>3 years) did not correlate with the concentration of CAR+ cells in the blood of patients on days 28-56, but did correlate with higher expansion and peak cell concentration on days 6-17 (70). These data suggest that a therapeutic window of 3 months with persisting 19-28ζ cells was sufficient to achieve a complete response.

## 5 Dual costimulation of CAR-T cells

Since the CD28 and 4-1BB domains signal via different pathways, a number of researchers have sought to clarify whether combining the two costimulatory domains in a single CAR might provide synergistic costimulation, resulting in increased expansion and elimination of tumor mass (characteristic of 19-28ζ), and increased durability (characteristic of 19-28ζ) (11). However, clinical trials are still controversial. In particular, a direct comparison was made between 2G and 3G CD19-specific CAR-T cells in the treatment of active lymphoma patients. The authors showed that the degree of expansion and persistence was increased by the inclusion of the 4-1BB domain in 19-28ζ (71). The effect was more pronounced in five patients with a low burden of disease and a low number of circulating normal B cells: 2G CAR-T cells had a limited expansion and persistence, whereas 3D CAR-T cells had a superior persistence.

However, the efficacy of 3G CARs has been shown to be inferior in a number of clinical studies. Namely, Abate-Daga D. et al. generated prostate stem cell antigen (PSCA)-directed CARs containing one or two costimulatory elements (72). In this study, the incorporation of the 4-1BB endodomain at the distal position to the cell membrane resulted in a reduction of the anti-tumor effect induced by CAR containing only the CD28 costimulatory domain. The phase I/IIa study of CD-19-targeted 3G CARs with the proximal CD28 domain and the distal 4-1BB domain in patients with B-cell lymphoma and leukemia (73), showed similar or even inferior results compared to 2G CARs (e.g. ZUMA-1, ref (63).). Complete responses were seen in 6 of 15 patients, with no apparent difference in durability compared to 2G CARs. The authors arrived into conclusion that the combination of CD28 and 4-1BB in the CAR may not lead to improve *in vivo* activity. It was not clear why there was no obvious synergy when two costimulatory receptors were combined in a single CAR.

The work of Muliaditan T. et al. provided some clarity in this matter (74). The authors designed a CAR construct in which CD28 and 4-1BB are positioned in trans within two separate receptors (2G (2G (CD28+CD3z) CAR was co-expressed with CAR consisting of antigen recognition domain and 4-1BB endodomain). This placed each of the costimulatory receptors in a more natural position - adjacent to the cell membrane. These CAR-T cells exhibited significantly greater target-dependent toxicity and proliferation after multiple cycles of re-stimulation (75) compared to 2G or 3G CARs with the same costimulatory domains. This CAR also showed superior anti-tumor activity (compared with 2G and 3G CARs) on a lymphoma xenograft mouse model, with markedly extended survival. Thereby, the researchers demonstrated that the synergetic effect of co-expressing CD28 and 41BB endodomains is achieved when both receptors are in a proximal position to the cell membrane. Clinical translation will be required to demonstrate the superiority of this approach over the 2G and 3G (with fused CD28 and 41BB endodomains) CAR-T cells in the treatment of both hematological malignancies and solid tumors.

## 6 Impact of CD4/8 cell balance and co-stimulation on CAR-T cell persistence

To date, CAR-T cells have mainly been administered at an undefined CD4/8 ratio. Nevertheless, there is clear evidence that costimulatory signals can affect CD4+ cells differently from CD8+ cells (76). For example, ICOS (inducible T cell co-stimulator (77)) costimulation plays an essential role in CD4+ T cell function (69). 4-1BB signaling is known to preferentially promote the survival of CD8+ T cells (70). However, recent work by Goodman D. et al. using polled CAR domains screening showed that under 4-1BB costimulation, CD4+ CAR-T cells expanded more than twice as much as CD8 CAR-T cells (78). In addition, CD4+ CAR-T cells with CD28 were shown to have a lower proliferation capacity than those with the 4-1BB domain in an *in vitro* repetitive stimulation assay. The role of costimulatory domains was investigated in separately cultured CD4+ and CD8+ T cells (79). The authors redirected them using CARs with different costimulatory domains (only activation with CD3ζ, CD28, 4-1BB, ICOS). CD4+ CAR-T cells with the ICOS costimulatory significantly increased the persistence of CD8+ (CD28 or 4-1BB) 2G CAR-T cells. The authors suggest that the combination of CD4+ CAR T cells with the ICOS costimulatory domain and CD8+ CAR T cells with the 4-1BB costimulatory domain is the best combination to increase the persistence of the CAR-T cell product.

In a recent report by Joseph Melenhorst, long-lasting CD19 redirected CAR-T cells with a 4-1BB costimulatory domain (CTL019) were studied in two patients with chronic lymphocytic leukemia (68). Using flow cytometry, the authors characterize CAR-T cells at different time points. Although CD8+ cells represent a prominent population at early time points (29.3% of CAR-T cells in patient 1 at month 1.8), CD4+ cells were the predominant CAR-T cell population at later time points (97.5% at year 1.4 and 99.6% from 3.4 to 9.3 years). A similar trend was observed in patient 2, with the exception of a prominent CD4- CD8- double negative CAR-T cell population (33.4% at month 2.5). By months 2.8 and 7.2 this population had decreased to 12.9% and 0.5% respectively. The CD4+ CAR-T cells had highly proliferative phenotype, with high expression of proliferation marker - Ki67, activation markers (CD38, CD95, HLA-D), memory markers (CD27, CCR7), as well as markers associated with bough activated and exhausted state (PD-1, TIM-3, LAG-3). Compared to their CAR- counterparts, CAR+CD4+ showed a strong up-regulation of the cytotoxic enzymes GZMK and GZMA. The authors concluded that although the initial response to CAR-T therapy is predominantly mediated by the CD8+ and double negative (CD4-CD8-Helios^hi^) population, at later time points a distinct population of CD4+ CAR-T cells is responsible for cytotoxicity against leukemia cells.

Taken together, the data from these studies highlight the key role of CD4+ cells in 19-BBζ persistence and suggest that separation of CD4+ and CD8+ cells and subsequent transduction of these cells with CARs bearing distinct costimulatory domains may enable longer persistence and improved effector function in the clinic.

## 7 Tonic signaling

As well as non-activated T-cells, CAR-T cells exhibit constitutive tonic signaling that can be defined as sustained activation in ligand independent manner (80, 81). Adrienne H. Long et al. demonstrated that a primary factor limiting the anti-tumor efficacy of CAR T cells is exhaustion, which is predicted by the structure and clustering of CAR receptors. Structure characteristics of extracellular antigen recognition domain appear to influence the magnitude of this signaling. For instance CAR targeting disialoganglioside GD2 exhibit strong tonic signaling and increases in exhaustion markers ex vivo (82). On the contrary, the most widely used CAR with scFv targeting CD19 based on FMC63 is not prone to significant tonic signaling (81). The authors showed that replacing the 19-28ζ scFv antigen-binding domain framework regions (regions that determine the structure of variable domain) with framework regions from the GD2 antigen recognition domain resulted in increased tonic signaling, which in turn led to rapid exhaustion of such cells (82). In addition, 4-1BB costimulatory receptors were reported to have the capacity to ameliorate 19-28ζ exhaustion (82).

Indeed, in the absence of structural support from IgG constant regions, the stability of scFv may be altered, rendering this domain susceptible to unfolding and aggregation (24). Furthermore, in a recent study of tonic signaling by 10 CARs differing only in the antigen recognition domain, the surface charge distribution was characterized. It was shown that CARs prone to tonic signaling had more positively charged residues on the surface of the antigen binding domain. These positively charged residues were the mediators of CAR clustering (83). This suggests that scFvs are key players in clustering and tonic signaling of CARs.

An immunoproteomic approach to characterize the CAR signalosome made by Maria C Ramello (84) identified that second generation CARs with CD28 costimulatory domain associated with an additional CD3ζ-containing protein that can be spontaneously phosphorylated. This observation provides additional possible reasons for tonic signaling and enhanced magnitude of activation in CD28 bearing CARs with consequent exhaustion at early stages of patient treatment.

## 8 Continuous stimulation of CARs under different co-stimulation conditions

Continuous stimulation with antigen or high level of CAR expression could cause the dysfunction of CAR-T cells that manifests as limited persistence, poor expansion and low cytotoxicity against tumor cells.

Persistent activation of T-cell leads to nuclear localization of NFAT with subsequent promotion of TOX and NR4A (NR4A1, NR4A2, NR4A3) expression that plays a central role in exhausted T-cell program commitment (85). CAR-T cells with triple knockout of three NR4A transcription factors prolonged the survival of tumor-bearing mice (86). Another work devoted to the investigation of exhausted CAR-T cells the with CD28 signaling domain, found the increased expression of AP-1/bZIP and bZIP/IRF transcription factors that have been implicated in regulation of exhaustion-related genes (87). Furthermore, the authors sought to investigate the role of the balance between activating and immunoregulatory functions of AP-1/IRF complexes in the onset of exhaustion states. In particular, over-expression of the AP-1 family transcription factor, which is linked to productive T-cell activation, c-Jun, enhanced homeostatic expansion and reduced the expression of exhaustion markers of CAR T-cells containing both the CD28 and 4-1BB costimulatory domains.

Chronic activation of CAR-T with either 4-1BB or CD28 domain by CD19+ acute lymphoblastic leukemia (ALL) cells was modeled by Selli et al. At day 15 both CAR-T cell products lost their ability to kill antigen-positive targets and could no longer make cytokines (88). 19-28ζ exhibited classical markers of T-cell exhaustion (PD-1, TIGIT, LAG3, TIM3, CTLA4). On the contrary, they didn’t observe classic exhaustion markers on 19-BBζ, which expressed higher levels of CD62L and CD25. Single-cell RNA sequencing revealed a high enrichment of exhaustion-associated genes in 19-28ζ, whereas 19-BBζ dysfunctional CAR-T cells were depleted of classical exhaustion signatures. The authors show that 19-BBζ reactivate the transcription factor FOXO3 in the exhausted state and that silencing FOXO3 reduces the onset of exhaustion in these cells by several days (88).

## 9 Additional stimulation with notch ligands

A variety of reports demonstrate that the sustained remission in patients with CLL correlates with the enrichment of memory related genes and enhanced quantities of stem cell–like memory T (TSCM) CAR-T cells (67, 89). Taisuke Kondo et al. managed to convert CAR-T cells into TSCM-like CAR-T via additional co-stimulation with NOTCH ligand dll1, expressed on OP9 cell line (90).

The CAR-T cells showed surface markers of a stem cell-like memory phenotype (CD45RA+CCR7+) and increased levels of memory-associated molecules (CD27, CD28 and CD62L) after the additional step of co-culturing with the OP9-hDLL1 cells. TSCM-like CAR-T cells were shown to almost completely eradicate leukemia cells in NSG mice, whereas leukemia cells persisted at high levels in mice treated with conventional CAR-T cells. Gene set enrichment analysis revealed that forkhead box protein M1 (FOXM1) underlies NOTCH-mediated iTSCM formation. The authors noted that FOXM1 regulates stemness, mitochondrial function and redox networks in various tumor cells (91, 92). The mitochondrial mass induced by co-culture with OP9-hDLL1 TSCM cells was decreased in FOXM1 deficient cells and increased in cells with FOXM1 over expression. Collectively, these data suggest a critical role for NOTCH-FOXM1 in mitochondrial biogenesis and induction of stem cell memory-like phenotypes in CAR-T cells, providing another modality to improve CAR-T cell persistence.

## 10 Discussion

CAR-T cells with both most frequently used costimulatory domains (19-28ζ or 19-BBζ) were shown to be effective in treating hematological malignancies using high doses of CAR-T products. However, in terms of activation amplitude and persistence, this efficacy is achieved in different ways. Clear differences in tumor elimination dynamics and persistence in a xenogeneic ALL model were only observed with reduced 19-28ζ and 19-BBζ doses. These findings are consistent with phosphoproteinomics data showing more intense phosphorylation of key proteins involved in T cell activation in 19-28ζ, and with clinical trials data showing more rapid expansion and clearance of tumor mass by 19-28ζ, and delayed onset of expansion (vs. 19-28ζ) but longer persistence of 19-BBζ.

In the context of low antigen expression on target cells and low dose of CAR-T cells, a stronger activating capacity and a lower activation threshold, achieved either by incorporating CD28 as a costimulatory domain or by incorporating part of CD28 as an H/TM domain with a 4-1BB costimulatory domain, seems to be advantageous. However, the use of 19-BBζ cells seems preferable from the point of view of potential off-target events when considering the targeting of antigens other than CD19, which are expressed both on cancer cells and, to a lesser extent, on healthy tissues.

Overall, the need to establish an appropriate balance between the amplitude of activation and persistence of CAR-T cells is strongly suggested by the data from preclinical and clinical studies using both CARs (19-28ζ and 19-BBζ). This balance could be achieved through the use of several strategies that have been shown to have the potential for modulation of CAR-T cell signaling in recent preclinical studies.

Indeed, it is possible to attenuate the activation of 19-28ζ by introducing mutations in ITEM within the CD3ζ moiety (1XX CAR), thereby increasing the persistence of 19-28ζ. On the other hand, increasing the number of ITAMs by adding an extra CD3ζ molecule to 19-BBζ (CD19-4-1BBζζ) increases its amplitude of activation, that enables eradication of tumors with low antigen densities. Finally, the combination of both costimulatory receptors in a single CAR-T product at their native site near the cell membrane has been shown to have synergistic effects. When two different antigen recognition domains with different costimulatory domains (e.g. 19-28ζ and 22-BBζ) are used, the strategy of dual costimulation seems particularly attractive. This approach allows us to combine the strengths of both costimulatory domains and reduce the likelihood of potential tumor recurrence due to loss of antigen expression.

In addition to new CAR designs, other strategies have been developed to increase the persistence of CAR-T cells. These include costimulation with NOTCH ligands during cultivation and genetic modifications such as triple knockout of NR4A transcription factors. Additional endurance and activation capacity can be achieved by combining these strategies with new CAR designs discussed below. To address this issue, however, further preclinical studies are required.

The novel strategies to modulate CAR-T signaling, which have shown excellent results in preclinical studies, are expected to offer new clinical modalities and contribute to extending the success of 2G CAR-T therapies in hematological malignancies to solid tumors. The 1XX CAR is currently in clinical trials.

## Author contributions

SS: Conceptualization, Writing – original draft, Writing – review & editing. PM: Writing – original draft. KS: Writing – original draft. ES: Writing – review & editing.
